# Integration of an Optical Ring Resonator Biosensor into a Self-Contained Microfluidic Cartridge with Active, Single-Shot Micropumps

**DOI:** 10.3390/mi7090153

**Published:** 2016-09-13

**Authors:** Sascha Geidel, Sergio Peransi Llopis, Manuel Rodrigo, Graciela de Diego-Castilla, Antonio Sousa, Jörg Nestler, Thomas Otto, Thomas Gessner, Victor Parro

**Affiliations:** 1 Fraunhofer Institute for Electronic Nanosystems (ENAS), Technologie-Campus 3, 09126 Chemnitz, Germany; thomas.otto@enas.fraunhofer.de (T.O.); thomas.gessner@enas.fraunhofer.de (T.G.); 2 DAS Photonics S.L., Valencia 46022, Spain; speransi@dasphotonics.com (S.P.L.); mrodrigo@dasphotonics.com (M.R.); 3 Centro de Astrobiología (INTA-CSIC), Torrejon de Ardoz 28850, Spain; diegocg@cab.inta-csic.es (G.D.-C.); parrogv@cab.inta-csic.es (V.P.); 4 Evoleo Technologies Lda, 442565 Maia, Portugal; antonio.sousa@evoleotech.com; 5 BiFlow Systems GmbH, 09126 Chemnitz, Germany; j.nestler@biflow-systems.com

**Keywords:** microfluidics, biosensor, optical ring resonator, micropumps, lab-on-a-chip

## Abstract

While there have been huge advances in the field of biosensors during the last decade, their integration into a microfluidic environment avoiding external tubing and pumping is still neglected. Herein, we show a new microfluidic design that integrates multiple reservoirs for reagent storage and single-use electrochemical pumps for time-controlled delivery of the liquids. The cartridge has been tested and validated with a silicon nitride-based photonic biosensor incorporating multiple optical ring resonators as sensing elements and an immunoassay as a potential target application. Based on experimental results obtained with a demonstration model, subcomponents were designed and existing protocols were adapted. The newly-designed microfluidic cartridges and photonic sensors were separately characterized on a technical basis and performed well. Afterwards, the sensor was functionalized for a protein detection. The microfluidic cartridge was loaded with the necessary assay reagents. The integrated pumps were programmed to drive the single process steps of an immunoassay. The prototype worked selectively, but only with a low sensitivity. Further work must be carried out to optimize biofunctionalization of the optical ring resonators and to have a more suitable flow velocity progression to enhance the system’s reproducibility.

## 1. Introduction

The standardization of biochemical analyses (e.g., diagnostic methods for virus detection, pregnancy or diabetes testing within medicine) lead to the demand for automation. A typical protocol for a biochemical analysis, or assay, consists (in the easiest setup) of a sequence of reagents put into contact with a sensitive area, which is selective to a target analyte. Since biosensors can be highly miniaturized, and necessary reagent volumes are within the microliter range, the tubes, valves, and actuators are the focus of miniaturization. The scientific field of microfluidics has developed many interesting actuation methods over the last decades [1,2,3,4,5,6]. Only a few of them are suited for disposable applications (necessary for contaminating samples), are scalable with respect to manufacturing counts, and integrate the necessary functions into a small package. The integrable electrolysis actuation method developed by Fraunhofer allows the integrated, automated assay processing with the just-mentioned boundary conditions [7,8]. The relevance of photonic-based systems in biosensing comes from the extremely good performance that those devices are able to show in terms of sensitivity (<10 ng/mL or 104 cell/mL), label-free detection, low reagent volume consumption (5–100 μL), multiplex detection, reusability, and ruggedness [9,10,11].

The efficient coupling of photonics and microfluidics is a current state of work within different scientific groups [12,13,14,15,16]. Currently, there is no published research which focuses on a photonic chip with multiple optical ring resonators for a multiplexed analysis in combination with a microfluidic cartridge that integrates liquid handling of all relevant liquids.

To achieve this goal a design for a microfluidic cartridge is accomplished. This cartridge integrates all necessary reagents for an immunoassay process. The actuation method is based on an integrated, microtechnological pumping method, which only needs minor external electronic infrastructure. A port within the microfluidic cartridge is designed to combine it with the photonic sensor. Within this first approach, lab-scale instruments are used to drive the laser and amplifier for the photonic sensor. The photonic sensor itself combines waveguides, grating couplers, power splitters, and ring resonators on one chip. The optical signal enters the photonic sensor through the alignment of an optical fiber array on top of the input couplers.

## 2. Materials and Methods

### 2.1. Microfluidic System

The materials and manufacturing processes used for the microfluidic cartridge have been described elsewhere [7,8]. Briefly, the fluidic structures were formed by injection molding of polycarbonate and were combined with a pumping layer stack. The pumping layer stack can be designed out of a number of independently working pumps. Each pump uses an electrolytic cell to generate gas within an electrolyte. The electrolytic cell is hermetically sealed and covered by a deformable membrane. The generated gas increases the pressure underneath the mentioned membrane, leading to a deflection of the membrane. A cavity within the fluidic substrate is placed on top of the electrolytic cell. A liquid stored within this cavity will be pushed towards an adjoining channel network.

Additional materials were used for the sealing ring, which forms the sensor chamber, and for the connection of tubes to the cartridge: the sealing ring is made out of Polydimethylsiloxane (PDMS, Sylgard^®^ 184 Silicone Elastomer Kit, Dow Corning Inc., Midland, MI, USA), which was cast. The mold for the casting was structured with a picosecond laser machine (microStruct vario, 3D Micromac AG, Chemnitz, Germany) into aluminum. To drive the pumps and to control the pumping sequence and flow velocity, commercially available software and electronics were used (flex.flow Eval-Kit, BiFlow Systems GmbH, Chemnitz, Germany). An adaptation of this kit was used for a first demonstration model, also.

The electrolysis reaction is (in the easiest setup) triggered by driving a constant current to an electrode, which is in contact with the electrolyte. The gas generation efficiency, the material characteristics of the cover membrane and the shape of the reagent cavity lead to a certain flow velocity progression of the pumped liquid. This characteristic curve was measured with a flow meter (Micro Flowmeter System for Liquids, GeSiM—Gesellschaft fuer Silizium-Mikrosysteme mbH, Großerkmannsdorf, Germany) for each pump.

### 2.2. Optical System

**Figure 1:**
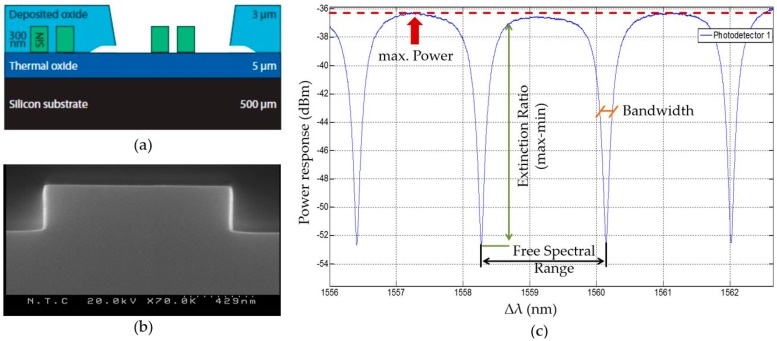
Manufacturing and characterization methodologies used for the fabrication of the photonic sensor: (**a**) cross-section showing the final layer structure of a photonic sensor with optical waveguides secured by an oxide and optical ring resonators in contact with the surrounding environment; (**b**) SEM picture of optical waveguides within silicon nitride. SEM images are done for technical characterization reasons of the single features by spot testing; and (**c**) response signal of an exemplary optical ring resonator. The included labels refer to central parameters describing the performance of each resonator.

The manufacturing of the photonic sensors is based on well-known complementary metal–oxide–semiconductor (CMOS) nanofabrication methods using silicon nitride as waveguides. Photonic waveguides at the scale of 1 µm for silicon nitride require very accurate fabrication. Imperfections in the nanometer scale (around 10 nm) lead to a large amount of scattering of power and, therefore, to propagation losses. The low tolerance of the technology is directly related to its integration capacity and sensitivity. The initial wafer is built out of a layer stack of silicon, silicon oxide, and silicon nitride. To avoid that the processed liquids for biochemical analysis are in contact with the optical structures, everything is covered with an oxide except from the optical ring resonators (Figure 1a). The ring resonators can be functionalized through that window, which will make them selectively sensitive for a target analyte. The process control was done through a scanning electron microscope (SEM) visualization method (Figure 1b), but mainly through optical performance measurements of the whole chip (Figure 1c).

The optical detection is based on a change of an optical signal within the sensing element, while matter is near its surface [17,18,19,20]. The optical ring resonator is located next to a waveguide guiding light of a certain wavelength. A power peak is induced into the response signal at certain frequencies of light by the optical ring resonators. At which wavelength the peak appears depends on the refractive index of the rings. A change of matter on the ring resonator leads to a change in the refractive index, which changes the wavelength of the power peak’s appearance [21,22]. The peaks are the optical feature that is tracked by the electronics. Theoretically, in the limit of shot noise, the detection limit for refractive indices is estimated to be in the order of 10^−9^ refractive index units. This makes optical ring resonator sensors sensitive enough for the detection of binding events of antibody bioreceptors to a target analyte.

The resonance characteristics of the sensor have to be validated regarding their performance (Figure 1c). Common parameters are *free spectral range*, *extinction ratio*, *bandwidth*, and *power*. The *free spectral range* is defined as the separation between two consecutive resonant wavelengths and has to be lower than the tuning range of the interrogating laser (lower than 100 nm) and larger than the absolute wavelength accuracy of 0.04 nm. At a resonance frequency, the signal lowers. The *extinction ratio* describes how large this drop is and the larger the signal drop the better the tracking of the signal can be. A value of more than 6 dB is considered to be of good performance. The *bandwidth* is defined as the frequency range of a resonance peak. It has to be as narrow as possible and a value of 100 pm is to be rated as a proper performance. The signal loses power while traveling through the system by scattering at the grating couplers or by splitting one signal up to multiple optical ring resonators. A maximum, absolute power difference of higher than 40 dB can be considered as performing well as long as the *extinction ratio* is large enough for an easily distinguishable resonance peak.

### 2.3. Micro-Ring Functionalization and Immunoassay Description

The optofluidic setup was tested with a biochemical assay, which makes biofunctionalization necessary. Therefore, the ring resonators were primed with epoxy groups. This pretreatment activated the rings chemically to allow a covalent binding of the antibody molecules [23]. The target analyte was the protein GroEL, which is universal in the prokaryotic microorganisms. One optical ring resonator was functionalized with a covalently-bound anti-GroEL antibody and another ring with the anti-cystein-BSA antibody. This last ring was used as a reference signal only. The concentration of the antibodies for functionalization was 0.8 mg·mL^−1^, while the target concentration of 1 µg·mL^−1^ of GroEL was use for label-free measurements. The test setup continuously reads out the sensor to discriminate between the levels of enhancement at which a significant signal increase can be observed. The initially label-free immunoassay was used with two additional signal enhancement stages. The first enhancement was done with a biotin-labeled secondary antibody (1 µg·mL^−1^ of biotinilated anti-GroEL) and the second enhancement with 1 µg·mL^−1^ streptavidin-coated nanoparticles.

The immunoassay process was derived from a standard laboratory process. Volumes and concentrations were adapted to be used with the microfluidic cartridge [24]. Table 1 lists the single steps of the assay procedure together with the volumes and the reagents used. Each liquid is supposed to be filled into a cavity within the microfluidic cartridge, which is connected to one of the integrated pumps. The sequence of pumps activated was programmed according to Table 1. Every reagent generates a signal shift within the sensor signal. After the first running buffer is flushed over the sensor, the egressing signal is set as the base lane of the assay signal progression (Table 1, step 1). To measure the bound target analyte all other components are washed away with the running buffer after the sample incubated the sensor (Table 1, step 2–3). The complexity of the prototype did not allow a prediction of the final sensitivity, which made it necessary to integrate signal enhancers. An antibody solution, to form a sandwich immunoassay (Table 1, step 4–5), and additional particles were used to amplify the signal if necessary (Table 1, step 6–7).

## 3. Results

This chapter shows the results obtained by building up an optofluidic setup for automatized immunoassay processing. To investigate a possible interfacing technique between the photonic integrated circuit (PIC) and the microfluidic cartridge, a demonstration model has been developed. A new microfluidic cartridge was designed based on volumes, assay steps, reagents, and interfacing necessities. The capability of the cartridge to pump stored liquids was characterized. The sensitivity of the photonic sensor was measured. To achieve selectivity, the photonic sensor was bio-functionalized to respond to the presence of a certain protein. The combined system of the microfluidic cartridge and photonic biosensor was tested during an exemplary immunoassay.

### 3.1. Demonstration Model

**Figure 2:**
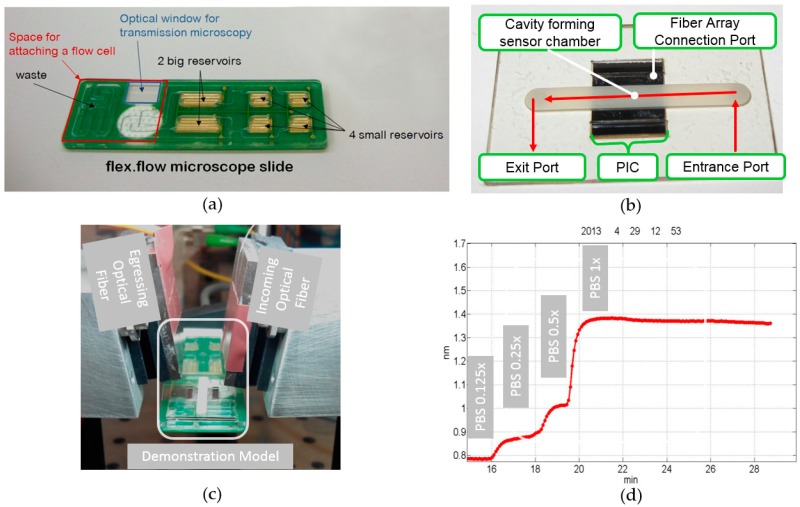
Demonstration model: (**a**) a standard flex.flow cartridge used for the study of the interfacing method between a microfluidic cartridge and the photonic sensor; (**b**) flow cell developed to form a sensor chamber on top of the optical ring resonators; (**c**) picture of the experimental setup with optical fibers aligned to the demonstration model; and (**d**) plot of the resonance frequency shift of the observed optical ring resonator, while different concentrations of phosphate buffered saline (PBS) are pumped through the sensor chamber.

The demonstration model is an adaptation of the commercially available flex.flow cartridge (BiFlow Systems GmbH) in combination with a photonic sensor prototype and a polymer foil layer stack. The flex.flow cartridge was used because it already utilizes the integrated actuation methodology. Since this technology will be part of the new design, it gave us the opportunity to early validating the sensor within a microfluidic environment. The combination of the standardized cartridge and the sensor was only possible because the cartridge has an open port for sensor applications. Figure 2a shows the cartridge and highlights the area for the sensor attachment with a red frame. There are two holes located, one coming from the six reservoirs and one descending into the waste.

The assembly of Figure 2b is positioned into the marked area of Figure 2a. It collects the liquid pumped from the reservoirs (Figure 2b, “Entrance Port”) and guides it through a channel to an exit port. This exit port is connected to the waste hole on the cartridge. In between the two ports the adapter has a cavity, where the photonic sensor is attached (Figure 2b, “PIC”). The adapter and the sensor are joined together by an adhesive layer. The adhesive layer ensures the liquid to be guided over the sensitive surface of the PIC and not leaking out of the system. A port for the coupling and decoupling of optical signals is kept open (Figure 2b, “Fiber Array Connection Port”). Optical fibers deliver the light from an external laser source to the sensor and transfer the decoupled light to the optoelectronic data acquisition unit (Figure 2c). The light exceeding the chip is recorded in situ and the sensor’s response is calculated from this data.

For a first test the six reservoirs of the cartridge were filled with varying concentrations of PBS and pumped sequentially one after another. The response signal of the photonic sensor was constantly monitored during the experiment. Figure 2d shows the shift of the resonance frequency of one observed optical ring resonator. The different PBS concentrations can be easily distinguished within the graph. For example, even the low concentration of 0.125 PBS was measured with a total resonance frequency shift of 0.07 nm. The experiment shows, that the volumes, flow rates, the dimensions of the sensor chamber, and the interfacing technique are suitable and can be used within a new cartridge design.

### 3.2. Design of a Microfluidic Cartridge

**Figure 3:**
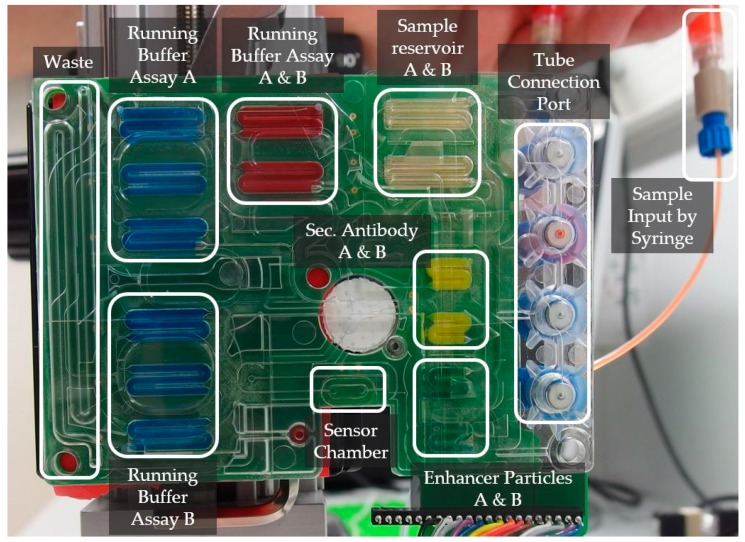
The picture of the newly-designed cartridge shows the reservoirs filled with colored water except the sample reservoirs. The sample is filled into the corresponding reservoirs using an external manipulator through tubing’s connected to a port with high performance liquid chromatography (HPLC)-fitting compatible threadings. The PIC is not shown here.

Figure 3 shows a cartridge specialized for the integration of the PIC and the immunoassay protocol described above. The different functional areas are labeled. The green surface indicates the integrated pumping capability. The color comes from the printed circuit board carrying the electrodes for each electrolysis cell. The functional elements used will be introduced starting from the sensor chamber located on the bottom edge of the cartridge: the sensor chamber is a cavity formed by a cast sealing ring made of PDMS, where the different reagents are pumped through to directly interact with the sensor surface. The oval-shaped sealing ring’s inner dimension is 2 mm in diameter and 6.5 mm in length. The sensor is fixed by force and aligned to this area through mechanical pins and a frame construction. The elastic characteristics, together with the mechanical clamping, leads to a clamping pressure dependency of the sensor chamber volume. The total height of the chamber was measured at different fixation forces, where no leakage occurred and the system was open for liquids to be pumped through. Heights between 0.07 mm and 0.19 mm were measured, which leads to a volume of the chamber between 0.8 µL and 2.3 µL.

The liquids flow through the sensor channel to a waste reservoir, which is structured into the fluidic substrate. The waste reservoir is large enough to take all the liquids, which are suppressed from their reservoirs by the pumping mechanism (650 µL). The liquids flowing into the waste would generate a pressure while compressing the air within it. To avoid this, the waste has a hole at the end, which sets the air free into the surrounding environment. The channel delivering the liquids to the sensor chamber arises from an area where the liquids are guided over a certain membrane (white circular-shaped element within the cartridge). This membrane works as a bubble trap: it is a filter membrane, which is hydrophobic and semipermeable for gasses. On top of the membrane, the liquids are getting pumped through a 125-µm thick channel, while gaseous phases are getting eliminated in between two subsequently pumped reservoirs. Without it, gaseous phases would enter the sensor area, which can hardly be removed once there, and which would disturb the signal. This membrane, and the hole at the end of the waste reservoir, form two necessary degassing elements within the system.

There are multiple channels entering the bubble trap membrane. They all come from one dedicated reagent reservoir and head towards one channel entering the sensor chamber. The channels entering the bubble trap form a network to connect all necessary reagent and sample reservoirs with the sensor area. The channel network generates a dead volume of 23 µL in total, but there is an additional dead volume coming from four holes, which will be used for valves later on, of about 13 µL. That means that one sample reservoir loses 5.6 µL while travelling from the reservoir to the sensor area. With respect to this dead volume, each reservoir was designed five microliters larger than specified by the assay. In total, there are ten reservoirs with 45 µL and four reservoirs with 25 µL of volume structured within the fluidic substrate. One reservoir is used for one assay step, but there are multiple reservoirs loaded with the same liquid. In Figure 3 three reservoirs are labeled with “Running Buffer Assay A” and are filled with the same reagent. The three reservoirs are not combined to one single reservoir because a once-started pumping process can hardly be interrupted. By stopping to power the electrolysis cell, the liquids will continuously flow for some time until the pressure within the system achieves an equilibrium state. At this point, the pumped volume is unknown and a newly-started pumping process would lead to an unpredictable behavior of the pump. Therefore, each assay step uses a separate reservoir, which will get pumped until the reservoir is totally empty. An emptied reservoir is defined to be completely filled by the membrane. This method has the side effect of ensuring a total flow velocity stop after each assay step as there is no remaining liquid to be pumped. To minimize the channel’s dead volume, channels were combined where possible. For example, reservoirs with the same reagent were positioned one after another and connected by one channel heading towards the sensor area.

The reagents are stored in the cartridge by directly pipetting the liquids into the reservoirs. The sample is loaded differently into the system: there are elements with threadings formed on top of the fluidic substrate. The ¼’’-28 UNF threading fits together with standard laboratory tube connectors. This interface allows external sample collector devices to load a sample into the system.

### 3.3. Design of the Photonic Sensor

**Figure 4:**
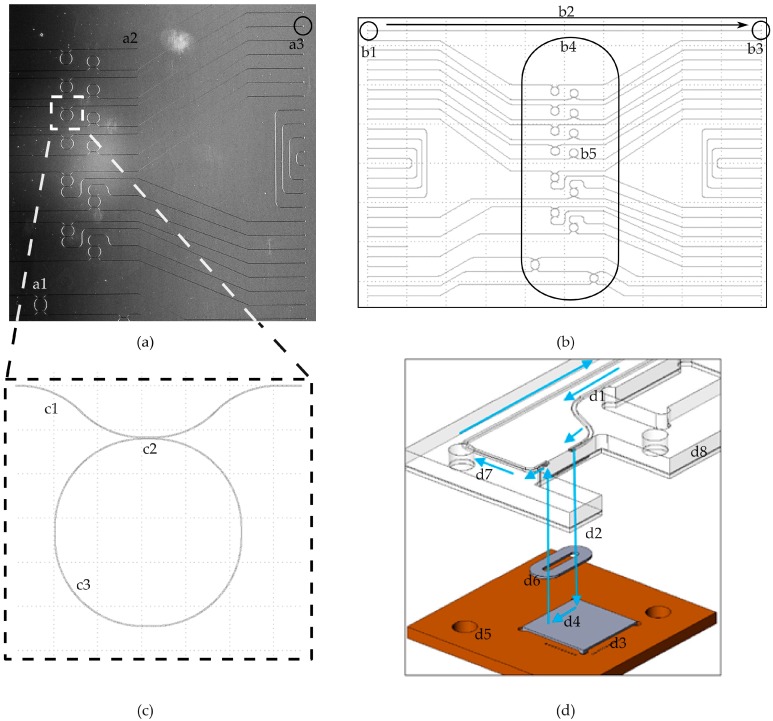
Design of the photonic sensor: (**a**) microscope image of a part of the photonic sensor with optical ring resonators (a1), optical waveguides (a2), and optical interfaces on the right (a3); (**b**) drawing of the whole circuit’s layout with an array of optical grating couplers on the left (b1) and on the right (b3) connected by a waveguide (b2). Optical ring resonators are located in the middle of the chip (b5). The black frame (b4) is not a part of the chip. It marks the area where the liquids will be pumped through; (**c**) detailed design of an optical ring resonator within the photonic sensor. An optical waveguide (c1) passes the optical ring resonator (c3) at a small area (c2); and (**d**) a schematic sketch of the fluidic connection of a polymer based cartridge (d8) and a silicon chip d4 using an elastomer sealing ring (d6). The direction of the pumped liquids is visualized by arrows.

The photonic circuit‘s layout outer dimension is 10 mm by 8 mm (Figure 4a,b). The coupling area is destined for the optical signal interlinking between an optical fiber array and the integrated waveguides (a2 and a3). The photonic sensor uses integrated optical interfaces known within the literature as grating couplers [9,10,11]. Despite the lower coupling efficiency vertical, grating couplers have been chosen for the following reasons: they are more robust to misalignment, are selective to the desired polarization (transversal, electric polarized light), and the vertical approach of the fiber allows a tighter and more robust assembly [25,26,27]. The couplers are marked with the labels b1 for the optical input and b3 for the output port, and are connected through a waveguide b2 (Figure 4b).

The flow cell area b4 is the photonic sensor’s section, where the liquids will be guided to by the microfluidic cartridge. It is 2 mm wide, 6.5 mm long, and within this area an array of sixteen optical ring resonators is located (b5). Figure 4c shows the layout of one ring resonator in detail: it has one waveguide c1, which guides the light past the ring c3. The material of the ring is not connected to the material of the waveguide, but optical signals passing by are influenced by the presence of the ring c2. Within a biochemical application, these sensing elements are differently functionalized to form a multiplexed biochemical sensor. The functionalization is done through bioreceptors (e.g., antibodies or aptamers), which are attached to the top of the ring resonators in order to interact with the target species flown on the sensing structures by means of a microfluidic channel.

The fluidic connection of the silicon-based sensor and the polymer-based fluidic cartridge is shown in Figure 4d. The direction of the pumped liquids is visualized by arrows. The liquids get pumped through a channel (d1) within a cartridge (d8). The channel ends at a hole, where the liquids get pushed out of the cartridge (d2) into the cavity formed by the sealing ring (d6). The liquids flow down the cavity of the sealing ring (d4) over the surface of the sensor (d3). Afterwards, they are pumped back into the cartridge through another hole and guided within a channel (d7) to a waste reservoir or an outlet port. Both components are aligned through holes (d5) by pins of an additional clamping device.

### 3.4. Technical Validation on Component and System Level

#### 3.4.1. Performance of the Pumps

**Figure 5:**
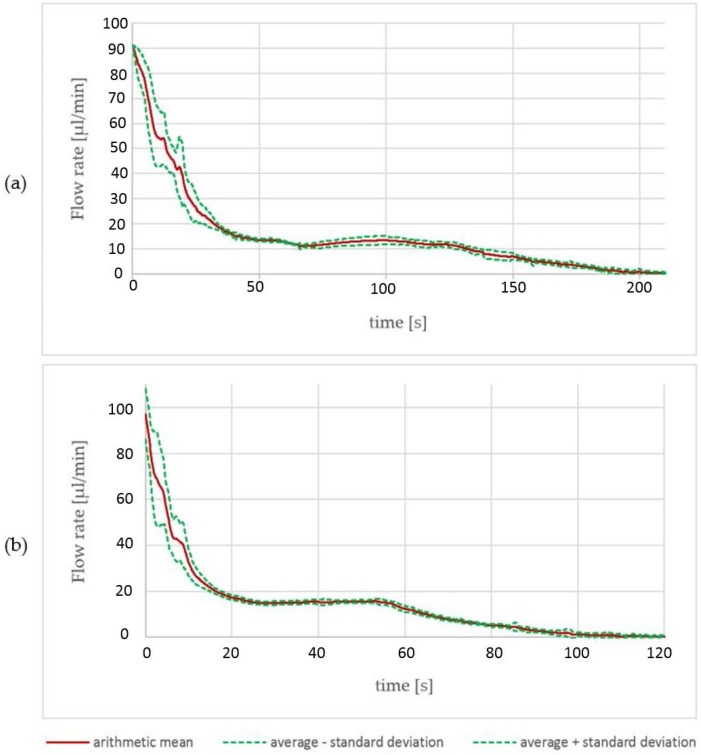
Data achieved by the pump characterization process of (**a**) 45 µL reservoirs and (**b**) 25 µL reservoirs. Each graph shows the arithmetic mean of the measured flow velocity achieved by an electrolysis reaction. The failure tube is formed by the standard deviation of 13 reservoirs with a volume of 25 µL and eight reservoirs with a volume of 45 µL.

The results obtained by the characterization process of the pumps are depicted in Figure 5. There are two pumps with different reagent volumes within the cartridge. Thirteen reservoirs with a volume of 25 µL, and eight reservoirs with a volume of 45 µL, were investigated. A current source was used to drive a constant current into the electrolysis cell. While pumping, the water within the cavity on top of the electrolysis cell was pushed through a tube towards the flow velocity sensor. The data of multiple pumps was collected and average values regarding pumped volume and flow velocity progression were calculated.

For the 45 µL pumps, a pumped volume with an average of 46.6 µL was measured. At the beginning of the pumping process, there are high values of the standard deviation. Until 40 s of pumped time, there is a maximum standard deviation of 13.1 µL/min and a minimum of 0.74 µL/s (Figure 5a). Afterwards, the standard deviation decreases to values between 1.8 µL/s and 0.2 µL/s. For the 25 µL pumps, comparable data was collected: the actual average volume was 27.6 µL and the flow rate showed a standard deviation of between 20.3 µL/min and 2.1 µL/min in the first 20 s and values between 2.0 µL/s and 0.1 µL/s for the remaining pumping time. The peak standard deviation of 20.3 µL/min is 50% higher than the data obtained from the 45 µL reservoir type. The high peak standard deviation values come mainly from a pump-dependent gradient of the flow velocity drop. With a decreasing gradient, the standard deviation decreases progressively.

With a current of 8 mA, the 45 µL reservoirs were empty after a pumping time of 200 s and the 25 µL reservoirs after 100 s. We did not observe bursts of the membrane or other malfunction. The time to empty the reservoir is a rough estimation derived from all data. At this point, we have not defined a reservoir empty criteria. Suitable criterions could be time, flow velocity, or volume.

#### 3.4.2. Performance of the PIC

The photonic sensor was characterized regarding its technical performance with no bio-functionalization done so far. The sensing elements are characterized by adjusting fibers to the photonic sensor through two alignment stages. The alignment stages use a closed loop control with a piezo actuator and a strain gauge to position the fibers over the optical input and output ports of the sensor. The power meter sweeps the laser through the observed spectra and a digital-to-analog converter records the movement of the resonance frequency of the optical ring resonators.

**Figure 6:**
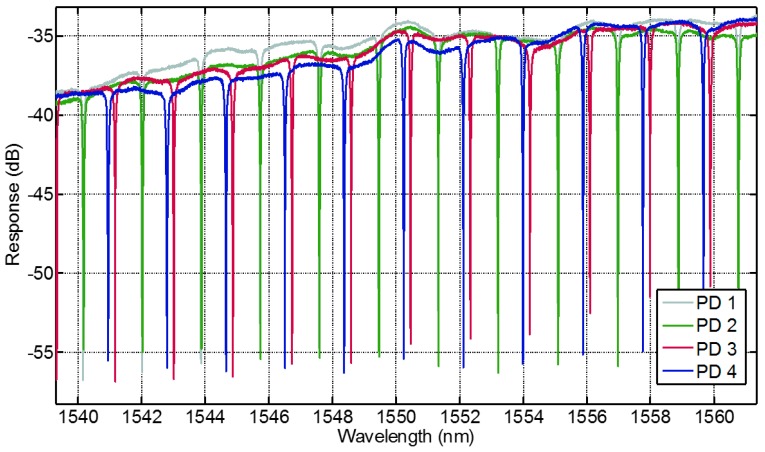
Measurements taken by the photonic sensor. Depicted is the response of four different optical ring resonators of one photonic sensor. The response signals are well defined and each separate frequency shift of one optical ring resonator can be easily tracked.

Figure 6 shows four typical response signals. The extinction ratio of all obtained curves is better than 10 dB making it feasible for peak detection. Both bandwidth (<100 pm) and the maximum power response (between 34 dB and 38 dB) are within a suitable range. The average free spectral range is 2 nm, which makes the tracking of signal shifts in a range of ±0.5 nm easily manageable. Since the absolute wavelength accuracy is ±0.04 nm, signal shifts in the range of ±460 pm can easily be distinguished. Imperfections throughout the fabrication process lead to differences in the individual power transmission of the rings. Otherwise this issue does not impede a proper implementation of the sensor if appropriate spectra calibration is taken before the use of a sensor.

#### 3.4.3. Optofluidic Immunoassay

For the immunoassay experiment, the microfluidic cartridge was joined together with the photonic sensor. A pumping sequence of the reagents derived from the immunoassay protocol was programmed using a current source with a multiplexer. The flow velocity itself was not controlled, but each electrolysis cell was driven with a constant current of 8 mA. Accordingly, the final flow velocity of each pump followed the progression measured and shown in Section 3.4.1. The shift of the resonance frequency of each ring was constantly monitored.

**Figure 7:**
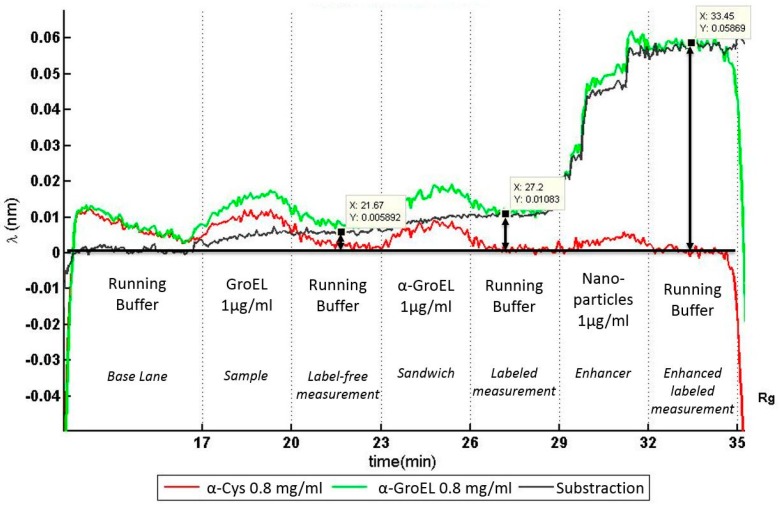
Optofluidic immunoassay. Results of an immunoassay experiment automatically performed by the microfluidic cartridge and monitored by the photonic sensor. With a differential frequency shift of 60 pm, only the labeled measurement, which was enhanced with functionalized nanoparticles, showed a significant signal increase.

Figure 7 shows the real-time, in situ monitored results of the automatically performed immunoassay process. As described before, the photonic response of two rings was recorded: one functionalized with anti-GroEL (green) and another with the reference anti-Cystein (red). The difference between both signals is shown in black and marks the selective capture of GroEL protein.

The differential resonance frequency increases starting from the sample introduction at minute 17. The label-free measurement (*t* = 20 to 23 min) shows a small signal corresponding to an average signal shift of 5 pm, which is lower than the accuracy of the laser source of 40 pm. To enhance the response signal from the sensor, a secondary antibody, anti-GroEL labeled with biotin, was flushed over the sensor surface. This generated a shift of the differential resonance frequency of 10 pm. Eventually, a second enhancement system with streptavidin-coated nanoparticles (streptavidin is a protein that specifically binds to biotin) clearly increased the resonance signal to a differential resonance frequency shift of 60 pm; higher than the accuracy of the laser.

## 4. Discussion

The results obtained from the technical characterization of the microfluidic subcomponent and the photonic subcomponent were promising. The pumps showed a comparable behavior without malfunction and only small deviation. The single sensing elements of the photonic sensor performed well, although shifts within the characteristics of single rings were observed. Here the fabrication tolerances did not allow a more matching picture, but are not crucial due to the differential measurement characteristic. Parameters, like extinction ratio, bandwidth, and the free spectral range showed good performance. Their values were in a range where the shift of the resonance frequencies should easily be tracked during a biochemical experiment.

The first biochemical experiment with an integrated optofluidic prototype for biosensing applications was able to perform an automatized immunoassay at this early stage. However, the concentration of the target analyte was quite high, only qualitative results were obtained, and it was necessary to add sandwich antibodies and signal-enhancing nanoparticles.

Further developments should show a concept for constant flow rate control. The microfluidic cartridge itself can be tuned to achieve an optimized incubation of the different reagents on the sensor and to avoid a possible cross-talk between the single assay steps. The incubation scheme of reagents on the sensor surface is different from a laboratory scale process. The incubation process takes place within the sensor chamber; a space with a volume between 0.8 µL and 2.3 µL (depending on the adjustable height of the chamber). The reagent can rest or flow over the sensor surface. This allows a multi-parameter optimization process for each single assay step. The results may indicate that lower volumes, or even fewer process steps, are possible. As a consequence, a new design of the cartridge could be even smaller leading to a more affordable solution. However, the design introduced here already integrates components which were not used for this investigation so far, but can be used in the future. An automatized sample introduction using integrated valves, as well as reusability investigations using the additional reservoirs for a second assay, can be performed.

The fluidic interface to the photonics seams to play a major role for the overall performance. A not totally symmetrical alignment of the sensing element array within the flow could lead to different incubation times due to the laminar flow characteristics. A different layout of the sensing elements can lead to a larger usable sensitive surface and to a higher optical coupling efficiency at the corresponding waveguide of each ring resonator. Integrated photodiodes can lead to better performance due to the avoidance of unnecessary optical coupling losses. Optimization tasks within the bio-functionalization section cannot be discussed in depth here. The significant drop in sensitivity of the sensor can come from unselective binding somewhere within the cartridge, or selective binding events may happen at the functionalized optical ring resonators, where they are not able to sense the binding anymore because it happened too far away from the ring.

## 5. Conclusions

This work describes a multidisciplinary approach, where highly-integrated microfluidics were combined with a sophisticated photonic sensor suitable to allow protein measurements with a one-step analytic device. The developed photonic sensor incorporates an array of optical ring resonators for biological assays with potentially high significance for multi-analyte samples. The overall design of the photonic sensor was adjusted to meet the fluidic interface of the microfluidic cartridge. The sensor was functionalized to show selectivity against a target analyte. Therefore, antibodies were covalently bound to one ring, which made it selective for the target protein (GroEL) and another ring was functionalized differently, serving as a blank reference.

The microfluidic cartridge was loaded with the different reagents for the immunoassay by utilizing cavities within the injection molded part of the cartridge. Underneath these cavities single-shot micropumps were integrated, which can be independently driven by an external current source setup. A special cartridge design was done to meet all requirements, especially electrical, optical, and mechanical interfaces, for an integration of the photonic sensor, of all biochemical reagents and process steps. The newly-developed microfluidic and photonic systems were technically characterized. Both systems were combined for an automated, nanoparticle-enhanced, sandwich immunoassay. The different liquids for each assay step were pumped by the microfluidic cartridge. The integrated photonic sensor was able to monitor the process and notice the presence of each assay liquid. A significant signal increase was measured after the last step of the assay. This showed a selective capture of the target analyte by the biofunctionalized layer.

The investigation showed, that photonics and microfluidics can be combined to form a highly-integrated device for biochemical analysis. Future work has to be done to evaluate the performance of the assay, which leads to an optimized design of the microfluidics and the photonics. The amplification of the sensitivity is a major goal. For a better usability of the system, storage methods, reduction of reagent payload, reduction of cartridge dimension, and exchangeability issues have to be investigated. By achieving the goals a miniaturized system can be developed using an affordable, small, and low-power-consuming stack of electronic and optic infrastructure, despite the use of laboratory equipment.

## Figures and Tables

**Table 1:** Description of the assay procedure. The single steps will be performed automatically by the microfluidic cartridge and monitored by the integrated photonic sensor. Each step defines solutions and volumes of the reagents to be loaded into the microfluidic cartridge.

Function	Solution	Volume (µL)
Base lane	Running Buffer	40
Sample	GroEl-Sample	40
Washing	Running Buffer	40
Secondary Antibody	α-GroEl	20
Washing	Running Buffer	40
Nanoparticle Enhancer	Nanoparticle solution	20
Binding lane	Running Buffer	40
